# Transition From a Paper Diary to an Electronic Diary by Parents of Preschool Children With Food Allergies: Pilot Study

**DOI:** 10.2196/73400

**Published:** 2025-10-21

**Authors:** Yoriko Kato

**Affiliations:** 1 School of Nursing, Sapporo City University, North 11 West 13, Chuo-ku, Sapporo, Hokkaido, 060-0011, Japan, 81 (11) 7262500

**Keywords:** electronic diary, food allergies, paper diary, parents, preschool children

## Abstract

**Background:**

Food allergies (FAs) can be life-threatening and represent a significant public health concern. The reported prevalence of FA in Japan is 7.6% in 1-year-olds, 6.7% in 2-year-olds, and 4.9% in 3-year-olds. In addition, avoiding allergenic foods, prompt identification, and response are required when symptoms appear. A food diary is a tool for daily dietary management, recording of symptoms, and communication between families of children with FA and health care providers. Conventionally, paper diaries (P-diaries) have been used. Recent technological advancements and the extensive use of smartphones have prompted the development of numerous electronic diaries (E-diaries). However, few studies have evaluated the effectiveness of E-diaries in Japan.

**Objective:**

This study aimed to assess the experiences of parents of preschool children with FA as they transitioned from P-diaries to E-diaries.

**Methods:**

For 6 months commencing in October 2020, during the period of movement restrictions due to the COVID-19 pandemic, we recruited parents of preschool children diagnosed with FAs by an allergist at a single general hospital. This pilot study used a single-group pretest–posttest design to evaluate the feasibility of using an E-diary. To assess parents’ views on transitioning to E-diaries from P-diaries, data were collected using semistructured interviews conducted after 6 months of E-diary use.

**Results:**

Five parents took part in the study. The problems with P-diaries and the effects and outcomes of E-diaries from the perspective of parents of preschool children with FA were categorized into 52 codes, which were grouped into 19 subcategories and 6 categories. Parents of preschool children with FA reported the following issues: *I. difficulties with using a P-diary*; *II. stress related to sharing information with doctors*; *III. difficulties in making disease management habitual as issues with P-diary use*; *IV. ease of using the E-diary as an effect of the transition*; *V. more efficient sharing of information with doctors*; and *VI. improved adherence as outcomes of E-diary use*.

**Conclusions:**

E-diaries may resolve various issues associated with P-diaries and may be useful tools for communication between parents and physicians regarding disease management. Since the number of participants was small (ie, 5) and the study was conducted at a single facility, theoretical saturation was not reached. In the future, it will be necessary to increase the number of participants to validate these findings.

## Introduction

### Overview

Food allergies (FAs) are a public health problem [1] with a prevalence of 7.6% in 1-year-olds, 6.7% in 2-year-olds, and 4.9% in 3-year-olds in Japan [2]. FA is defined as “an adverse health effect arising from a specific immune response that occurs reproducibly on exposure to a given food” [3].

FA is a serious and life-threatening disease associated with anxiety, worry, and decreased quality of life for children and their families. The difficulty in avoiding causative foods and the unpredictable nature of FA-induced symptoms are factors affecting children with FA and their families. FA requires daily management and constant monitoring, and the chronic and unpredictable nature of FA may affect the psychosocial functioning and quality of life of the child and family [4].

Oral immunotherapy (OIT) is a promising treatment for FA [5]. Studies have shown that the completion of OIT or reaching its maintenance phase significantly improves the quality of life of both the child and parents [6,7]. However, OIT is a challenging process for patients and parents. Induction can generate stress and anxiety. This, in turn, may affect their motivation and ability to cope with OIT challenges [8]. OIT is administered over an extended period, ranging from several months to several years, and requires daily consumption of food that patients were taught to avoid and fear. Also, patients commonly have oral aversion to the allergenic food, making regular consumption more difficult [9]. Moreover, adverse reactions are often experienced during treatment, ranging from mild symptoms to anaphylaxis [5].

The occurrence of frequent adverse reactions poses a challenge, necessitating close monitoring during treatment. OITcontrol (University of Navarra, Pamplona, Spain) appears to be a valuable tool for monitoring treatment in children with FAs. It is a suitable method for recording daily intake and reactions, promoting adherence to treatment instructions following adverse events, as well as compliance with dose adjustments for home reactions [10].

eHealth monitoring technologies offer opportunities to objectively assess symptoms when they appear in daily life [11]. The convenience and accessibility offered by digital health technologies in health care have the potential to transform the health care landscape by enhancing communication, improving efficiency, and empowering patients [12]. In addition, the use of patient-centered digital health records in nonhospitalized individuals with chronic health conditions is potentially associated with considerable benefits for health care use, treatment adherence, and self-management or self-efficacy [13]. However, limited research has been conducted on the use of electronic diaries (E-diaries) for FA.

Retrospective questionnaires have been used for decades to assess the severity and control of allergic diseases. Smartphone apps have recently facilitated the use of prospective clinical diaries based on daily patient-completed questionnaires [14].

FA diaries record the types and amounts of food and drink consumed, as well as the types and severity of induced symptoms. E-diaries may make it easier for parents to inform doctors of FA-induced symptoms by using the camera function on their smartphones, particularly when maintaining records of cutaneous symptoms. Most food diaries for FA in Japan are paper-based, and no confirmed experience exists with the use of electronic food diaries by parents.

A mobile health (mHealth) app (provided by Mylan EPD), released in Japan in September 2020, aimed to provide accurate medical information on FA and anaphylaxis, as well as to encourage the proper use of an adjuvant anaphylaxis drug (adrenaline self-injection).

### Purpose

This study aimed to assess the experiences of parents of preschool children diagnosed with FA as they transitioned from a paper diary (P-diary) to an E-diary, and to evaluate their perceptions of the technology.

## Methods

### Study Design

This pilot study used a quasi-experimental one-group pretest-posttest design to evaluate the E-diary technology. To investigate the parents’ views on transitioning from regular P-diaries to E-diaries, the authors used data obtained from semistructured interviews conducted 6 months postusage of E-diaries to evaluate their usefulness compared to that of P-diaries.

### Participant Recruitment

Study participants who met the following criteria were recruited: (1) a parent of a preschool child receiving OIT and (2) someone with adequate information technology knowledge to use the app and transition from a P-dairy to an E-dairy using a smartphone. In addition, participants could send a PDF file summarizing the data of the E-diary to the researcher before the outpatient visit.

### Intervention Method

The E-diary used in this study was designed for recording content, including allergy symptoms, and saving photographs. It was part of the MyEpi mHealth app (provided by Mylan EPD), released in Japan in September 2020, which provides accurate medical information on FA and anaphylaxis and encourages the proper use of adjuvant drugs for anaphylaxis (adrenaline self-injection). Written approval for the use of the MyEpi mHealth app in this study was obtained from Mylan EPD.

### The MyEpi mHealth App Incorporates the Following 4 Functions

The MyEpi mHealth App includes the following 4 features:

1. Note function (E-diary): the user can record symptoms and test results, including photographs. These records can be printed or sent via email as PDFs.
2. FA library function: provides the user with accurate information on matters, including the mechanism of allergies and appropriate responses in case of anaphylaxis.
3. Q&A function: users can access answers to frequently asked questions on topics such as the causes, symptoms, and treatment of allergies.
4. Emergency voice navigation: in the event of anaphylaxis, the user can use this function to follow step-by-step video and voice instructions for administering the adjuvant anaphylaxis drug.

### The Investigators Requested Study Participants Who Provided Informed Consent to Perform the Following 3 Actions

**Figure 1.**
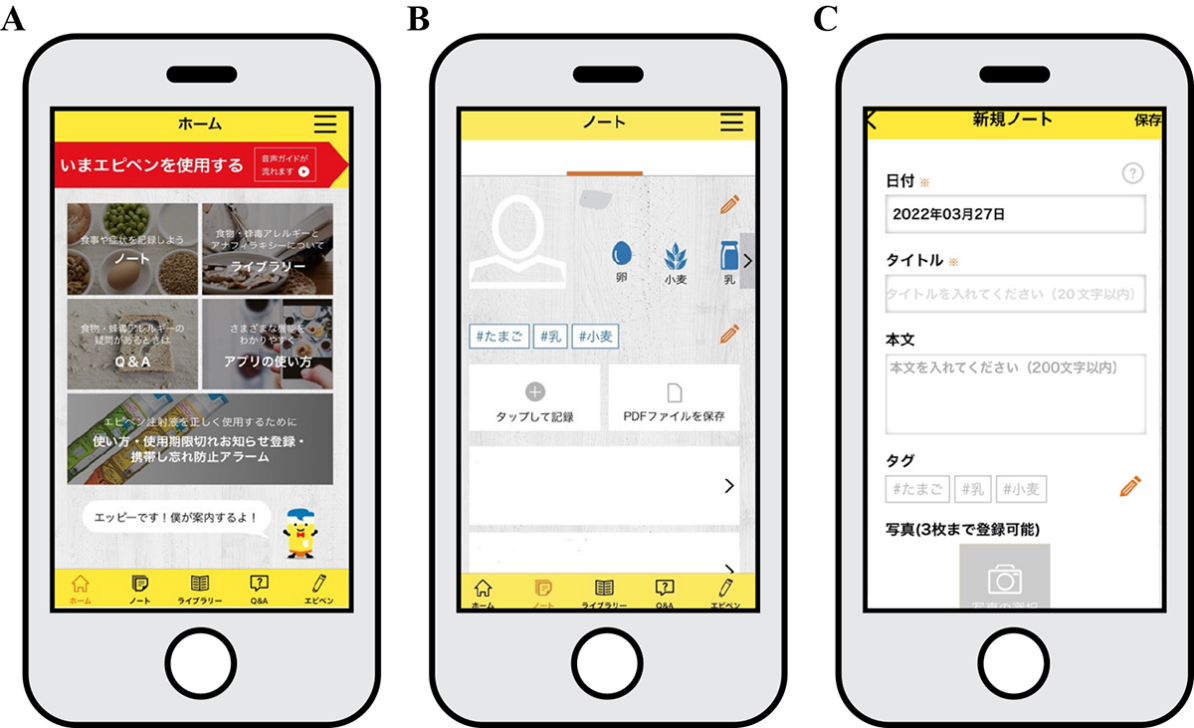
Overview of the MyEpi app. Source: Mylan EPD.

These 3 actions are given below:

1. Download the MyEpi mHealth app and explore its use by following the Operation Manual, which provides simple instructions on how to input E-diary entries and save photographs.
2. After trying out the app, attach the E-diary entries and photographs as PDFs to an email and send it to an investigator’s personal email address to confirm operational feasibility.
3. Send the PDF data to the study email address 1 week before the scheduled outpatient appointment (Figure 1).

The investigators were responsible for forwarding the E-diary PDF data, emailed by the study participants, to the doctors.

### Data Collection

To evaluate the E-diary after its introduction, the authors used data obtained from semistructured interviews conducted 6 months postusage of the E-diary to evaluate its usefulness compared with that of the P-diary. An interview guide was prepared in accordance with the study objectives. The investigators provided the participants with the content of the interviews. All the interviews were conducted by the lead author, who had received training in conducting regular interviews. During each interview, the interviewer encouraged participants to speak freely about their experiences using the P-diary and E-diary. The interviewer did not assist participants with any information regarding FA. Demographic data on the child’s age, sex, age at onset of FA, and offending food were collected during the interview. The interview also covered participants’ experiences with (1) using the P-diary and (2) using the E-diary. With the participants’ consent, the interviews were recorded with a digital voice recorder.

### Data Analysis

We used Berelson “Content Analysis” method for data analysis [15]. “Content Analysis” is a method used for the objective, systematic identification of characteristics in text to draw inferences, ultimately enabling quantitative analysis [16]. All recorded data were transcribed verbatim in Japanese. Content related to (1) the experience of using a P-diary and (2) the experience of using an E-diary was coded as a unit. Subcategories and categories were generated based on the semantic similarity of the codes. The study team included 2 pediatric nurses (KY and KM). After the initial coding of each transcript, the investigators discussed the data together and identified a series of main codes, subcategories, and categories. Reliability was assessed during the coding stage, and a calculated concordance rate of ≥90% between the 2 investigators was confirmed. Validity was assured through the supervision of a pediatric nurse who served as a joint investigator.

### Ethical Considerations

This study was conducted with the approval of the Research Ethics Committee of the Faculty of Nursing and Social Work, Health Sciences University of Hokkaido (approval number 20N024030) and the Ethics Committee of the General Hospital.

The study participants who met the selection criteria were informed of the objective, importance, and methods of the study orally and in writing. The participants were informed by the investigators that they had the right to participate in the study of their own free will, including their right to withdraw at any time; that their data would be anonymized and confidentiality protected; how their data would be handled and disposed of; and the possibility of publication. Written informed consent was obtained. The study adhered to the COREQ (Consolidated Criteria for Reporting Qualitative Studies) guidelines [17]. Participants received a gift card worth 5000 Japanese yen as a research incentive.

## Results

### Participant Demographics

**Figure 2.**
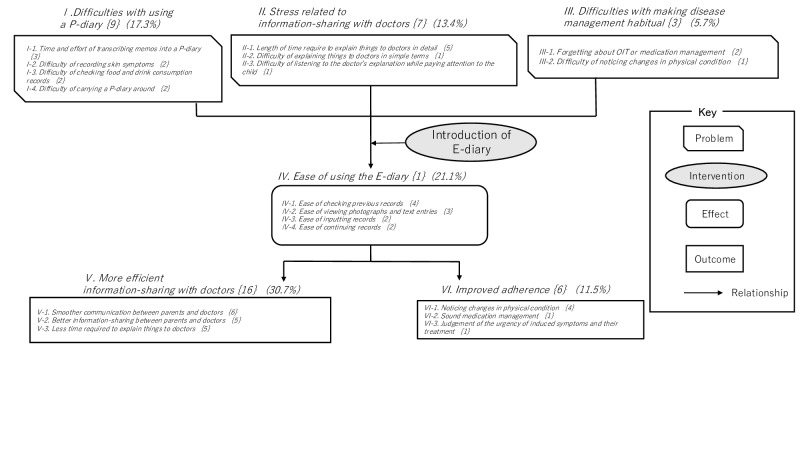
Problems associated with paper diary use and effects and outcomes of electronic diary use reported by parents of preschool children with food allergies. P-diary: paper diary; OIT: oral immunotherapy; E-diary: electronic diary.

All 5 parents who were contacted by telephone agreed to take part in the study. All were mothers in their 30s (Table 1). After using the mHealth app for 6 months, each participant underwent a single interview lasting between 26 minutes 40 seconds and 60 minutes 30 seconds (*M_time_*=40 min 29 s). These interviews were conducted between September and December 2021. Due to the COVID-19 pandemic, a single investigator specializing in childhood allergies conducted all the interviews via a videoconferencing system. The difficulties with P-diaries and the effects and outcomes of E-diaries were analyzed. The results of the analysis showed 52 codes were extracted, grouped into 19 subcategories and 6 categories. Categories are indicated in *italic* text, subcategories in *italic* text, code numbers in {curly brackets}, and percentages in (ordinary parentheses). The parents of preschool children with FA reported: I. difficulties with using a P-diary, II. stress related to information-sharing with doctors, and III. difficulties in making disease management habitual, as problems associated with the P-diary, while IV. ease of using the E-diary as a positive effect of the E-diary, V. more efficient information sharing with doctors, and VI. improved adherence, as favorable outcomes of the E-diary (Figure 2).

**Table 1.** Characteristics of children with food allergies whose parents continued using an electronic diary for 6 months (n=5).

Case	Ages at time of interview (years)	Sex	Age of onset (months)	Allergen
A	6 years 8 months	Boy	2	Wheat, Milk
B	3 years 10 months	Girl	6	Egg, Milk
C	6 years 1 month	Boy	5	Milk
D	6 years 5 months	Boy	11	Wheat, Milk
E	5 years 0 months	Girl	6	Milk

### Difficulties Associated With the P-Diary

Problems associated with the P-diary were categorized into 19 codes, which were grouped into 9 subcategories and 3 categories (Table 2).

**Table 2.** Problems associated with the paper diary, recognized by parents of children with food allergy after using the electronic diary for 6 months.

Categories and subcategories		Representative codes	Codes
(I).Difficulties with using^a^ P-diary			9 (17.3%)
	I-1. Time and effort of transcribing memos into a P-diary	*Copying the records, I’d noted on a calendar into a memo was twice as much trouble.* [Case C, 30 s, mother of a 6-year-old boy] *Before I started using the electronic food diary, I’d be rushing to organize the information to tell the doctor.* [Case E: 30 s, mother of a 5-year-old girl] *Recording symptoms and daily behavior together took double the time and effort.* [Case E, 30 s, mother of a 5-year-old girl]	3
	I-2. Difficulty in recording skin symptoms	*It’s difficult to explain the condition of roughened skin to the doctor in words.* [Case A, 30 s, mother of a 6-year-old boy] *The subtle skin symptoms, where the skin appears red and swollen around the mouth, ears, and navel, are difficult to record.* [Case E, 30 s, mother of a 5-year-old girl]	2
	I-3. Difficulty in checking food and drink consumption records	*If there are several offending foods, they have to be eaten on different days, which means comparing the records for each offending food one by one.* [Case B, 30 s, mother of a 3-year-old girl] *The electronic food diary enables me to check several different offending foods at once without getting them mixed up.* [Case B, 30 s, mother of a 3-year-old girl]	2
	I-4. Difficulty in carrying a P-diary around	*If I’m using a paper-based medium for the food diary, the paper and notes get scattered and lost.* [Case A, 30 s, mother of a 6-year-old boy] *When we went on holiday, it was very hard to take the paper-based food diary with us.* [Case A, 30 s, mother of a 6-year-old boy]	2
(II).Stress related to information-sharing with doctors			7 (13.4%)
	II-1. Length of time required to explain things to doctors in detail	*I’m always concerned about time during appointments.* [Case B, 30 s, mother of a 3-year-old girl] *It was time-consuming for the doctor to input the electronic medical records, too, and it seemed like a lot of trouble.* [Case C, 30 s, mother of a 6-year-old boy] *I used to give a detailed oral report to the doctor of everything that had happened since the last appointment.* [Case C, 30s, mother of a 6-year-old boy] *At appointments, I would give the doctor in charge the records of the offending food recorded in a schedule planner, and give a detailed explanation while looking at the schedule planner.* [Case D, 30 s, mother of a 6-year-old boy] *Before using the electronic food diary, I used to give a detailed explanation.* [Case E, 30 s, mother of a 6-year-old girl]	5
	II-2. Difficulty in explaining things to doctors in simple terms	*I’m not good at conversation, so I can’t explain things clearly to the doctor in charge, and my way of recording things also didn’t work well, so I would get really antsy at appointments.* [Case A, 30 s, mother of a 6-year-old boy]	1
	II-3. Difficulty in listening to the doctor’s explanation while paying attention to the child	*I would pay attention to my son while talking to the doctor, so they didn’t believe everything I said.* [Case C, 30 s, mother of a 6-year-old boy]	1
(III). Difficulties with making disease management habitual			3 (5.7%)
	III-1. Forgetting about OIT^b^ or medication management	*When I was using a paper-based food diary, if I didn’t put it somewhere I could see it, I’d forget about OIT.* [Case A, 30 s, mother of a 6-year-old boy] *If one of my children were taking medication, I’d forget to give medication to the other one.* [Case C, 30 s, mother of a 6-year-old boy]	2
	III-2. Difficulty in noticing changes in physical condition	*He had a tendency to get sick at the changing of the seasons.* [Case C, 30 s, mother of a 6-year-old boy]	1

a P-diary: paper diary.

b OIT: oral immunotherapy.

### Effects of the E-Diary

Aspects of the effects of the introduction of the E-diary were categorized 11 codes, which were grouped into 4 subcategories and 1 category (Table 3).

**Table 3.** Effects of paper diary recognized by parents of children with food allergy after using electronic diary for 6 months.

Categories and subcategories		Representative codes	Codes
(IV).Ease of using the E-diary^a^			11 (21.1%)
	IV-1. Ease of checking previous records	*The electronic food diary made it easier to check the amount of the offending food consumed and the frequency of consumption.* [Case A, 30 s, mother of a 6-year-old boy] *The electronic food diary is convenient because you can view the previous history whenever you want.* [Case B, 30 s, mother of a 3-year-old girl] *The electronic food diary enables me to check several different offending foods at once without getting them mixed up.* [Case B, 30 s, mother of a 3-year-old girl] *The electronic food diary makes it easy to look back over the course of symptoms.* [Case C, 30 s, mother of a 6-year-old boy]	4
	IV-2. Ease of viewing photographs and text entries	*Text entries and photographs can be recorded in one go, and when they are printed out, the entries and photographs also come out together.* [Case E, 30 s, mother of a 5-year-old girl] *The electronic food diary enables the unified management of records and photographs of symptoms.* [Case C, 30 s, mother of a 6-year-old boy] *It was convenient to capture photographs as well as descriptions of what sort of symptoms had been induced and when.* [Case B, 30 s, mother of a 3-year-old girl]	3
	IV-3. Ease of inputting records	*My smartphone is small enough to carry around the whole time, so I no longer go looking for the food diary.* [Case A, 30 s, mother of a 6-year-old boy] *The electronic food diary makes it easy to input anything that has worried me, and the details of how I dealt with symptoms when they appeared.* [Case A, 30 s, mother of a 6-year-old boy]	2
	IV-4. Ease of continuing records	*The electronic food diary has lowered the hurdles for record-keeping.* [Case A, 30 s, mother of a 6-year-old boy] *The double time and effort involved in record-keeping disappeared.* [Case C, 30 s, mother of a 6-year-old boy]	2

a E-diary: electronic diary.

### Outcomes of the E-Diary

Aspects of the outcomes of the E-diary were categorized into 22 codes, which were grouped into 6 subcategories and 2 categories (Table 4).

**Table 4.** Outcomes of paper diary recognized by parents of children with food allergy after using electronic diary for 6 months.

Categories and subcategories		Representative codes	Codes
(V).More efficient information-sharing with doctors			16 (30.7%)
	V-1. Smoother verbal communication between parents and doctors	*Since the doctor in charge had already looked at the data before the appointment, the conversation during the appointment was smooth.* [Case A, 30 s, mother of a 6-year-old boy] *Since the doctor in charge had already finished checking the data in advance, the examination was focused on key points, and the conversation went quickly.* [Case C, 30 s, mother of a 6-year-old boy] *Using the electronic food diary meant that the explanation during the appointment went smoothly.* [Case E, 30 s, mother of a 6-year-old girl] *The doctor operates the tablet and enlarges the photographs of skin symptoms, which makes it easier to explain them to the doctor.* [Case A, 30 s, mother of a 6-year-old boy] *It’s difficult to explain the condition of roughened skin to the doctor in words, but photographs are easier to explain to the doctor.* [Case A, 30 s, mother of a 6-year-old boy] *The electronic food diary has made the conversation with the doctor in charge go smoothly, and enabled the consultation to be pinpointed.* [Case C, 30 s, mother of a 6-year-old boy]	6
	V-2. Better information-sharing between parents and doctors	*Since the doctor in charge has already received the data before the appointment, the conversation can move forward.* [Case B, 30 s, mother of a 3-year-old girl] *The electronic food diary was useful during telephone conversations and outpatient appointments.* [Case B, 30 s, mother of a 3-year-old girl] *The stress of telephone appointments has actually decreased.* [Case B, 30 s, mother of a 3-year-old girl] *I send the electronic food diary data to the doctor in charge before appointments.* [Case C, 30 s, mother of a 6-year-old boy] *It was useful to convey the electronic food diary data to the doctor in charge.* [Case E, 30 s, mother of a 6-year-old girl]	5
	V-3. Less time required to explain things to doctors	*Using the electronic food diary has given us more time during appointments, enabling me to listen carefully to the doctor in charge.* [Case E, 30 s, mother of a 6-year-old girl] *I’ve become able to make full use of the time during consultations.* [Case B, 30 s, mother of a 3-year-old girl] *Examinations used to last around 20min but now finish in 10min.* [Case C, 30 s, mother of a 6-year-old boy] *The introduction of the electronic food diary has reduced the time it takes to explain what had happened.* [Case D, 30 s, mother of a 6-year-old boy] *The time taken to explain things to the doctor in charge during appointments has come down to around 10min.* [Case E, 30 s, mother of a 6-year-old girl]	5
(VI).Improved adherence			6 (11.5%)
	VI-1. Noticing changes in physical condition	*Continuous use of the electronic food diary has made symptoms easier to predict.* [Case A, 30 s, mother of a 6-year-old boy] *Above all, I’m now able to notice changes in his physical condition after consumption, following the doctor’s instructions.* [Case A, 30 s, mother of a 6-year-old boy] *By continuing the electronic food diary, I am now able to analyze changes in his physical condition in each season.* [Case C, 30 s, mother of a 6-year-old boy] *Because it took 3 y to become used to the paper-based food diary, I have accumulated experience until now, and have come to understand how to predict induced symptoms.* [Case D, 30 s, mother of a 6-year-old boy]	4
	VI-2. Sound medication management	*When inputting oral medication into the electronic food diary, I can recollect the times I forgot to give medication.* [Case C, 30 s, mother of a 6-year-old boy]	1
	VI-3. Judgment of the urgency of induced symptoms and their treatment	*I remembered that the doctor in charge had told me that I could come to my own judgment on reducing the frequency of inhalation if symptoms improved, and was able to undertake treatment at home without making a mistake.* [Case C, 30 s, mother of a 6-year-old boy]	1

## Discussion

### Principal Results

Parents of preschool children with FA reported the following: *I. difficulties with using a P-diary***,** *II. stress related to sharing of information with doctors***,** and *III. difficulties with making disease management habitual* as problems with the P-diary; *IV. ease of using the E-diary* as an effect; *V. more efficient sharing of information with doctors;* and *VI. improved adherence* as outcomes of E-diary use.

### Electronic Diary Transition Experiences by Parents of Preschoolers With FA

We explored the experiences of parents of preschool children with FA during the transition from a P-diary to an E-diary.

During the transition, parents reported categories I, II, and III as issues with the P-diary, category IV as an effect of E-diary use, and categories V and VI as outcomes of E-diary use.

#### Difficulties With the P-Diary

Categories I, II, and III were problems associated with the P-diary. The first problem was *I. difficulties with using a P-diary*. In a study evaluating the use of P-diaries for pain in participants with chronic pain, 90% (36/40) reported compliance with completing the P-diary. However, when the actual time of the diary entry was monitored electronically, true compliance was 11%. Hoarding was common with the paper diary [18]. Many patients recorded in their diaries just before returning them—known as “parking lot entries” [19]. This may be inferred from the large number of codes related to *I-1. time and effort required to transcribe memos into a P-diary*.

In addition, the participants cited *I-2. difficulty recording skin symptoms*, *I-3. difficulty checking food and drink consumption records*, and *I-4. difficulty carrying a P-diary around*. A previous study [20] reported that “skin symptoms were the most common immediate allergic reaction to FA, occurring in 92% of patients, followed by respiratory symptoms in 33.6%, mucous membrane symptoms in 28%, and digestive symptoms in 18.6%. The severity of symptoms of immediate allergic reactions due to FA should be evaluated in each organ and treated accordingly.” Visual information on skin symptoms, such as erythema, urticaria, and wheals, is difficult to express verbally. The difficulty in verbally describing skin symptoms may have contributed to the difficulty in recording them, which includes not only food and drink consumption but also the consumption of snacks, eating out at restaurants, and the occurrence of symptoms in their temporal context. The type and intensity of symptoms are recorded with the date of occurrence and, if necessary, the duration. The use of drugs is also recorded [21]. This complex record content may have contributed to the difficulty in checking FA intake records.

With respect to *II-1. the length of time required to explain details to doctors* and *II-2. the difficulty of explaining information to doctors in simple terms*, before the introduction of the E-diary, no opportunity existed for parents and doctors to share information about the contents of the P-diary before outpatient visits. This may have increased the time required to explain progress in detail to the doctor during the visit, leading to stress related to the sharing of information. The participant also mentioned *II-3. difficulty listening to the doctor’s explanation while attending to the child*. Health care providers need to communicate effectively to explain all treatment options, including continued causal food avoidance and OIT [22]. However, another study [23] showed that parents often receive far more information during a pediatric visit than they can realistically remember. The study also noted that environmental distractions of having young children nearby during office visits may further impair memory. These findings are consistent with the present results.

The third problem was *III. difficulties with making disease management a habit*. Participants cited *III-1. forgetting about OIT or medication management*. OIT starts below the threshold value (the minimum amount that would elicit a reaction), with the oral dose gradually increasing over time to improve clinical tolerance of the target food. Since this treatment entails a few risks, parents of children diagnosed with FA must be equipped to manage induced symptoms [22]. Inadvertently forgetting OIT is a serious problem for maintaining FA treatment. A previous study [18] reported that with P-diaries, 75% (n=5) of patients accumulate a day’s worth of records. This accumulation of records is called “parking lot entries” [19]. With P-diaries, it has been suggested that OIT and medication administration may be forgotten, leading to difficulty in disease management. In addition, participants indicated *III-2. difficulty noticing changes in physical condition*. Diaries generally capture changes in symptoms over time [24].

Diaries documenting FA-related diet and symptoms are useful for providing patients and their families with a more concrete understanding of their lifestyle and symptoms. However, with P-diaries, it was suggested that difficulties in using the diary may have hindered the detection of changes in physical condition.

#### Effects of the E-Diary

Category IV addressed the effects of the E-diary. Participants cited *IV-1. ease of checking previous records* as one subcategory. An E-diary is a powerful and efficient tool for collecting complex self-reported data. It provides unique insights into the symptom experience by documenting significant changes [25]. Patients reported that the greatest advantage of E-diaries as a tool was the ease of reviewing the course of symptoms. The participants also mentioned *IV-2. ease of viewing photographs and text entries*. Previous studies have reported the value of smartphone apps for monitoring visual information on cutaneous lesions in atopic dermatitis using photos [26] and for the daily evaluation and recording of atopic dermatitis symptom scores [27]. Another study suggested that smartphone apps may be effective in improving patients’ awareness and management of anaphylaxis [28]. These findings may provide new insights into the effectiveness of FA-related E-diary use. In addition, participants cited *IV-3. ease of inputting records* and *IV-4. ease of continuing records*. Since smartphones are easy to use anytime and anywhere, they enable the effective collection of real-time symptoms and their sharing with medical professionals, which has been reported as useful for monitoring cutaneous symptoms of atopic dermatitis [27]. The present results are consistent with this view, suggesting that the introduction of the E-diary may help resolve problems associated with the P-diary.

#### Outcomes of the E-Diary

Categories V and VI represented the outcomes of the E-diary. The first outcome was *V. more efficient information-sharing with doctors*. Participants mentioned *V-1. smoother verbal communication between parents and doctors*. Diaries that include visual cutaneous symptoms have been reported to be useful for doctors for diagnosis [26], as well as for helping parents to convey their child’s symptoms accurately. Participants also cited *V-2. better information-sharing between parents and doctors*. According to a previous study [29], parents want improved information-sharing and communication with health care providers. They reported that communication technology has the potential to improve communication and coordination of care. The present results are consistent with this view. In addition, the participants cited *V-3. less time required to explain information to doctors*. In a survey of parents’ perceptions of the use of mHealth apps, 71% (n=5) reported that it saved time for doctors and nurses [26]. The present results are consistent with this view.

The second outcome was *VI. improved adherence*. The participants cited *VI-1. noticing changes in physical condition*. The goal of FA care is to help patients and their caregivers manage the risk of food allergic reactions, reduce food-related anxiety, and gain a sense of control over their condition [22]. To enable them to acquire this sense, if the patient is a young child, the doctor must instruct the parents to record the amount of the offending food consumed and the allergic symptoms induced. Diaries are generally considered effective in capturing symptom variability over time [24]. The results indicate that the E-diary may be useful as an efficient recording tool. The participants also indicated *VI-2. sound medication management*. Numerous mobile phone medication adherence apps are presently available and may serve as useful tools to enable patients to take medication as prescribed [30]. The present results are consistent with this view.

In addition, the participants listed *VI-3. judgment of the urgency of induced symptoms and their treatment*. A previous study [23] showed that during pediatric visits, parents often receive far more information than they can realistically remember. Such information, which often covers multiple broad areas (eg, disease complications and treatments), is associated with poorer memory compared to information on narrower topics. These results represent a novel outcome of the use of an FA-related E-diary.

### Limitations

This study had some limitations. After using the E-diary for 6 months, 5 parents were asked about their experience transitioning from the P-diary. Since only 5 parents were interviewed at a single medical institution, some of the aspects evaluated may have been underestimated or overestimated. In addition, the allergens of children with FA were milk, wheat, and eggs—the 3 major allergens in Japan—representing a potential selection bias. However, factors such as the number of years from diagnosis to interview, severity of FA, and number of allergens—which may affect how parents use the E-diary—were not considered in the selection of participants.

### Strengths and Future Challenges

This study was the first in Japan to confirm the use of an E-diary specifically designed for parents of children diagnosed with FA. The results improve our understanding of the use of E-diaries by parents of preschool children with FA and have important practical implications for patient education.

Future studies should include a larger sample size and a longer follow-up period (eg, 1 year). Notably, all participants in this study were mothers. In Japan, despite the increasing participation of fathers in childcare, mothers typically record information in the E-diary and accompany their children to outpatient appointments. However, future surveys should include both fathers and mothers.

In Japan, few reports exist on the use of E-diaries. In this study, to facilitate sharing the contents of E-diaries between parents and physicians, the researchers received data in PDF format from the parents and then forwarded it to the physicians. The presence of the researcher as an intermediary was a positive factor in the use of the E-diary. The role of the researcher has not been fully evaluated. Further research is needed on the role of intermediaries who support medical treatment using E-diaries.

### Role of Supporters Linking Parents and Doctor

During the first 6 months after the introduction of the E-diary, the researcher was responsible for converting the data recorded in the E-diary into PDF files and sending them to the physician before the outpatient visit. van der Kamp et al [31] reported that communication, as demonstrated by self-monitoring data, builds trust between parents, children, and health care providers and enhances understanding and self-management of illness. The environment in which parents and physicians could share the contents of the E-diary prior to the visit may have facilitated effective communication. Sezgin et al [29] pointed out that a systematic approach using communication technology is needed to enable parents to share information with their health care providers. Furthermore, it has been reported [32] that when promoting eHealth interventions, it is important to have specific opinion leaders in the hospital who can encourage the adoption of digital care. These pilot study findings are important for the promotion of E-diary–based medical care. To facilitate a systematic approach in the future, it is necessary to secure supporters of E-diary implementation.

### Conclusions

The authors found that parents of preschool children with FA were aware of the following problems with P-diaries: *I. difficulties with using a P-diary*; *II. stress related to information-sharing with doctors*; and *III. difficulties with making disease management habitual*. They reported *IV. ease of using the E-diary* as an effect of its use. They further reported *V. more efficient information-sharing with doctors* and *VI. improved adherence* as outcomes of using the E-diary. E-diaries will contribute to solving the problems of P-diaries and may be a useful tool for communication between parents and physicians, as well as for disease management.

## Supplementary material

10.2196/73400 Checklist 1 COREQ checklist.

## Abbreviations

COREQ: Consolidated Criteria for Reporting Qualitative Studies
E-diary: electronic diary
FA: food allergy
mHealth: mobile health
OIT: oral immunotherapy
P-diary: paper diary
