# Correction: Transition From a Paper Diary to an Electronic Diary by Parents of Preschool Children With Food Allergies: Pilot Study

**DOI:** 10.2196/92855

**Published:** 2026-02-26

**Authors:** Yoriko Kato

**Affiliations:** 1 School of Nursing, Sapporo City University, North 11 West 13, Chuo-ku, Sapporo, Hokkaido, 060-0011, Japan, 81 (11) 7262500

In “Transition From a Paper Diary to an Electronic Diary by Parents of Preschool Children With Food Allergies: Pilot Study” [1], the author made one correction.

In the Introduction section, the following sentence was revised:

The OITcontrol app (Mylan EPD) appears to be a valuable tool for monitoring treatment in children with FAs.

This sentence now reads:

OITcontrol (University of Navarra, Pamplona, Spain) appears to be a valuable tool for monitoring treatment in children with FAs.

The correction will appear in the online version of the paper on the JMIR Publications website, together with the publication of this correction notice. Because this was made after submission to PubMed, PubMed Central, and other full-text repositories, the corrected article has also been resubmitted to those repositories.
